# Placental growth factor silencing ameliorates liver fibrosis and angiogenesis and inhibits activation of hepatic stellate cells in a murine model of chronic liver disease

**DOI:** 10.1111/jcmm.13158

**Published:** 2017-04-05

**Authors:** Xi Li, Qun‐Yan Yao, Hong‐Chun Liu, Qian‐Wen Jin, Bei‐Li Xu, Shun‐Cai Zhang, Chuan‐Tao Tu

**Affiliations:** ^1^ Department of Geriatrics Zhongshan Hospital Fudan University Shanghai China; ^2^ Department of Gastroenterology and Hepatology Zhongshan Hospital Fudan University and Shanghai Institute of Liver Diseases Shanghai China

**Keywords:** hepatic fibrosis, cirrhosis, placental growth factor, angiogenesis, small interfering RNA, hepatic stellate cells

## Abstract

Placental growth factor (PlGF) is a member of the vascular endothelial growth factor (VEGF) family and is involved in pathological angiogenesis associated with chronic liver diseases. However, the precise mechanisms underlying PlGF signalling contributing to liver fibrosis and angiogenesis remain largely unexplored. This study aimed to assess the effect of reducing PlGF expression using small interfering RNA (siRNA) on experimental liver fibrosis and angiogenesis, and to elucidate the underlying molecular mechanisms. Fibrosis was induced in mice by carbon tetrachloride (CCl_4_) for 8 weeks, and mice were treated with PlGF siRNA or non‐targeting control siRNA starting two weeks after initiating CCl_4_ injections. The results showed that PlGF was highly expressed in cirrhotic human and mice livers; which mainly distributed in activated hepatic stellate cells (HSCs). PlGF silencing robustly reduced liver inflammation, fibrosis, intrahepatic macrophage recruitment, and inhibited the activation of HSCs *in vivo*. Moreover, PlGF siRNA‐treated fibrotic mice showed diminished hepatic microvessel density and angiogenic factors, such as hypoxia‐inducible factor‐1α (HIF‐1α), VEGF and VEGF receptor‐1. Moreover, down‐regulation of PlGF with siRNA in HSCs inhibited the activation and proliferation of HSCs. Mechanistically, overexpression of PlGF in activated HSCs was induced by hypoxia dependent on HIF‐1α, and PlGF induces HSC activation and proliferation *via* activation the phosphatidylinositol 3‐kinase (PI3K)/Akt signalling pathways. These findings indicate that PlGF plays an important role in liver fibrosis‐associated angiogenesis and that blockage of PlGF could be an effective strategy for chronic liver disease.

## Introduction

Liver fibrogenesis is a complex dynamic wound‐healing response in the liver and is defined by the accumulation of excess extracellular matrix (ECM) deposition 1, 2, 3. Activated hepatic stellate cells (HSCs) are believed to be the main ECM‐producing cells with the injured liver and thus responsible for the development of liver fibrosis 1, 2, 3. In response to injury, HSCs become activated and migrate to the sites of tissue repair, secreting large amount of ECM and regulating ECM remodelling 1, 2. Therefore, the activation of HSCs is the key event in the liver fibrogenesis 1, 2, 3. Although understanding of the mechanisms underlying the pathogenesis of liver fibrosis has increased, there is currently no established pharmacological treatment for this disorder 3, 4. Thus, it is important to thoroughly analyse the pathologic mechanisms associated with liver fibrosis in order to find newly targets for antifibrotic therapies.

Recently, emerging evidence suggests that there is a strong link between pathological angiogenesis and the formation of liver fibrosis 5, 6, 7, 8, 9. It is well known that angiogenesis is controlled in a large part by the balance between pro‐angiogenic growth factors and a diverse group of endogenous inhibitors of angiogenesis 5, 6, 10. Among molecules involved in angiogenesis, vascular endothelial growth factor (VEGF) is one of the most well‐characterized angiogenic factors, which plays an important role in liver fibrogenesis and angiogenesis 5, 6, 7, 8, 9, 10. Moreover, most of the studies have demonstrated that therapeutic targeting angiogenesis such as blocking VEGF receptor (VEGFR) signalling pathway, blockade Toll‐like receptor 4 and adenovirus expressing the extracellular domain of Tie2 inhibited liver fibrosis 9, 10, 11, 12. In addition, hepatic inflammation may be served as a process linking angiogenesis and fibrogenesis 10, 11, 12. It therefore has been shown that multitargeted therapies acting against both angiogenesis and inflammation are beneficial in inhibiting the progression of fibrosis to cirrhosis 5, 6, 7, 8. However, VEGF is a trophic factor for healthy vessels, and therefore, anti‐angiogenic therapies cause side effects 13. Moreover, several studies reported that inhibition of angiogenesis could worsen fibrogenesis in specific conditions 14, 15, 16. Notably, recent studies have also demonstrated that VEGF promoted fibrosis resolution and repair in rodent models 15, 16. Therefore, further understanding the mechanisms regulating angiogenesis is essential to develop new therapeutic strategies that specifically target pathological angiogenesis without affecting physiological angiogenesis and fibrosis resolution 8, 15, 16, 17.

Placental growth factor (PlGF) is a member of the VEGF family and is involved in bone marrow‐derived cell activation, endothelial stimulation, inflammation, pathologic angiogenesis and wound healing 13, 17, 18, 19, 20. PlGF is overexpressed in cirrhotic liver and hepatocellular carcinoma (HCC) both in human and in rodent models 17, 21, 22, 23, 24. Unlike VEGF, PlGF is dispensable for development and health 13, 17, 18, 19; thus, blockage of PlGF pathway has been shown to reduce pathological angiogenesis in various spontaneous cancers and other disease models without affecting healthy blood vessels 13, 17, 18, 19. Recent studies have indicated that blockade of PlGF by antibody or genetic ablation in animal model reduced fibrogenesis and portal hypertension and inhibited HCC 21, 22, 23, 24.

However, the precise mechanisms underlying PlGF signalling contributing to liver fibrosis and angiogenesis remain largely unexplored. Therefore, the aims of the study were to evaluate the role of PlGF in liver fibrosis and angiogenesis using small interfering RNA (siRNA) technology, and to provide mechanistic insight into the fibrogenic role of PlGF by demonstrating its biological effect on HSCs *in vivo*.

## Materials and methods

### Regents and antibodies

The following reagents were used in this study: carbon tetrachloride (CCl_4_), 4‐hydroxy‐L‐proline, Nycodenz, type IV collagenase, olive oil and LY294002 were from Sigma Chemical, Co. Ltd (St Louis, MO, USA). Foetal bovine serum (FBS), trypsin, Dulbecco's modified Eagle's medium (DMEM), penicillin and streptomycin were from Gibco (Carlsbad, CA, USA). Lipofectamine^®^ 2000, Invivofectamine^®^ 2.0 Reagent, *in Vivo Silencer* ^®^ Select Pre‐designed and Validated PlGF siRNA and *in vivo* non‐targeting control (NTC) siRNA were from Life Technologies (Carlsbad, CA, USA). Recombinant rat PlGF was from ReliaTech GmbH (Wolfenbüttel, Germany). Recombinant human PlGF protein and human PlGF Quantikine ELISA kit were from R&D Systems Inc. (Minneapolis, MN, USA). Cell Counting Kit‐8 (CCK‐8) assay kit was from Yeasen Biotech Company (Shanghai, China).

Antibodies were purchased as indicated: mouse anti‐α‐SMA and rabbit anti‐von Willebrand factor (vWF) (Dako North American, Inc. Carpentaria, CA, USA); rat anti‐PlGF, rabbit anti‐PlGF, collagen type III, CD31, CD34, F4/80, CCL2, VEGF, VEGFR‐1, VEGFR‐2, NRP‐1, ICAM‐1, mouse anti‐SMA and HIF‐1α (Abcam, Cambridge, CA, USA); mouse anti‐CXCL10 (R&D Systems Inc., Minneapolis, MN, USA); rabbit anti‐GAPDH, β‐actin, β‐tubulin, PI3K, Akt and p‐Akt (Cell Signaling Technology, Boston, MA, USA); and HRP‐conjugated goat antimouse, rabbit and rat (Biotech Well, Shanghai, China).

### Animal experimental

**Figure 1:**
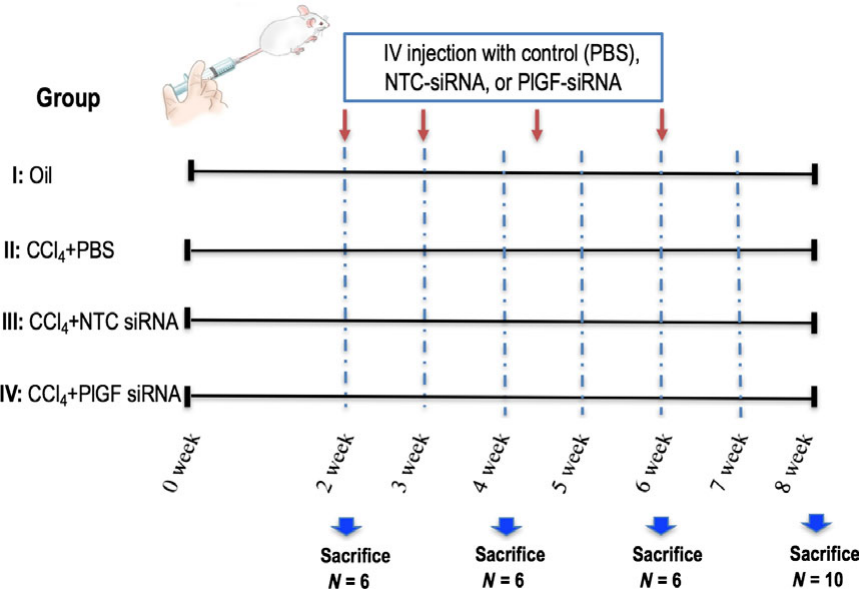
Experimental study design. Fibrosis was induced in mice by carbon tetrachloride (CCl_4_) for 8 weeks, and mice were treated with PlGF siRNA or non‐targeting control (NTC) siRNA four cycles *via* tail vein injection starting 2 weeks after initiating CCl_4_ injections.

Male BALB/c mice (7 weeks old, weighing 20–26 g) were purchased from Shanghai Laboratory Animal Research Center (Shanghai, China). The animals were kept in an environmentally controlled room (23 ± 2°C, 55 ± 10% humidity) with a 12‐hrs light/dark cycle and allowed free access to food and water. Liver fibrosis in mice was injected intraperitoneally (i.p.) with 0.5 μl/g of CCl_4_ diluted in olive oil twice a week for 8 weeks 25. Mice were randomly distributed in four groups as shown in experimental design (Fig. 1). To deliver each siRNA, *in vivo* ready siRNAs were mixed with Invivofectamine 2.0 regents and injected (i.p.) in a volume of 100 μl at a dose of 5 mg/kg for four cycles starting two weeks after initiating CCl_4_ injections. Six to 10 mice of each group were killed on weeks 2, 4, 6 and 8, respectively, and the liver was removed and cut into small pieces and either snap‐frozen in liquid nitrogen for storage at −80°C or fixed in freshly prepared 4% paraformaldehyde for 24 hrs at 4°C. Mouse sera were isolated to assay for liver functions. The study was performed in accordance with the guiding principles for the care and use of laboratory animals approved by the Fudan University Animal Care Committee.

### Human liver specimens

Human liver specimens were obtained from liver‐transplanted patients suffering from liver cirrhosis. Control tissue was obtained from the unaffected part of liver from transplanted patients in our hospital. All patients gave written informed consent in accordance with the Declaration of Helsinki, and the protocol, approved by ethical committees from the Hospital Clinic, and followed ethical guidelines on handling human samples.

### Isolation and culture of hepatic stellate cells

Primary HSCs were routinely isolated from normal male Sprague–Dawley rats by *in situ* enzymatic digestion of the liver with the pronase–collagenase method followed by a density centrifugation on a Nycodenz gradient as previously described 26. Freshly isolated rat HSCs were seeded into uncoated plastic dishes and grown in DMEM supplemented with 1.0 g/l glucose, 10% heat‐inactivated (56°C for 30 min.) FBS, 100 units/ml penicillin, and 100 mg/ml streptomycin in a humidified atmosphere of 5% CO_2_ at 37°C. The medium was changed every 2 days. The cells were subsequently digested with 0.25% trypsin once the cells had reached 80–90% confluence. Culture purity, assessed routinely by retinoid autofluorescence at 350 nm, was >95%.

### Cell treatment and transfection

LX‐2 human hepatic stellate cell line was from ScienCell Research Laboratory (Carlsbad, CA, USA) and was cultured as described previously 27. Primary rat HSCs were seeded into 6‐well culture plates 3 days prior to transfection. Once the cells had reached 70–80% confluence, they were separated into five groups and transfected with various siRNAs as follows: control group; siRNA1 group; siRNA2 group; and siRNA3 group. PlGF siRNA (siPlGF) and non‐targeted scrambled control siRNA (siNTC) lentiviral transduction particles were purchased from Genechem (Shanghai, China), and the synthesized oligos are shown in Table S1. Retroviral HIF‐1α shRNA (shHIF‐1α) and non‐targeting control shRNA (shNTC) were purchased from OriGene (Rockville, MD, USA). For transfection, PlGF siRNA (20 nM), HIF‐1α shRNA (50 nM) or appropriate control siRNA (or shRNA) was transfected into HSCs using Lipofectamine 2000 Regent. Knockdown efficiency was determined by quantitative RT‐PCR and Western blotting (Figs S1 and 7E–G). For some groups, rat HSCs were treated with rPlGF 48 hrs after shHIF‐1α transfection. In some experiments, cells were treated with different contents of rat or human rPlGF (0–100 ng/ml) in the absence or presence of the 10 μM LY294002 (a PI3K inhibitor) for 30 min. For another groups, cells culturing under hypoxic conditions (1% oxygen) were performed using an incubator with an oxygen concentration regulator system (Thermo Scientific, Waltham, MA, USA). Triplicate wells were established for each group.

For all experiments, rat HSCs or LX2 cells were incubated for an additional 24 hrs prior to immunofluorescence analysis, RNA harvesting and protein isolation.

### Cell proliferation

Cell proliferation assay was determined by CCK‐8 assay kit according to the manufacturer's instructions. Treated cells were incubated in 10% CCK‐8 that was diluted in normal culture medium at 37°C until the visual colour conversion occurred. Proliferation rate were measured at 450 nm absorbance with Flexstation 3 multimode microplate reader (Molecular Device, Sunnyvale, CA, USA). The experiments were conducted three times independently.

### Liver enzymes assays, hydroxyproline concentration and serum PlGF ELISA

Serum aspartate aminotransferase (AST) and alanine aminotransferase (ALT) concentrations were determined spectrophotometrically using an automatic biochemical analyser (Beckman, Fullerton, CA, USA). Hydroxyproline was measured in liver tissue hydrolysates using the Hydroxyproline Assay Kit (Milpitas, CA, USA) according to the manufacturer's instructions, and the results are expressed as a microgram of hydroxyproline per gram of liver tissue. Serum PlGF in patients with cirrhosis was measured by ELISA kit with Flexstation 3 multimode microplate reader (Molecular Device, Sunnyvale, CA, USA) according to the manufacturer's instructions.

### Histopathologic evaluation, immunohistochemistry and immunofluorescence

Liver tissues were fixed with 10% neutral buffered formalin, embedded in paraffin and cut into 5‐μm‐thick sections for histological and immunohistochemical analysis, and for H&E and Sirius red staining according to standard procedures. Fibrosis was quantified using ImageJ software (NIH, Bethesda, Maryland, USA) on 10 non‐contiguous Sirius red‐stained sections 7, 25 and by the Ishak modified histological activity index scoring system 7, 28. Portal inflammation was graded with a 0–3 scale as described previously 25, 28. A liver pathologist without knowledge of the treatment group examined histology. Protocol for immunohistochemistry and immunofluorescence is described in detail in the Appendix S1.

### Quantitative analysis of histological markers and angiogenesis

The number of α‐SMA‐positive cells and the intensity of collagen III immunostaining in tissue sections were quantified using five random non‐overlapping fields (×100) of each slide and determined for six animals in each group, and the area of staining was analysed as a percentage of the total area using the software NIH ImageJ 1.49 7, 25.

For quantification F4/80‐positive macrophages/Kupffer cells in liver sections, 10 microscopic fields (×400) were taken at random per liver section from each mouse, and all the macrophages included in the field were analysed. Five of each group were examined and then calculated 29, 30.

Microvascular density (MVD) in the liver tissue was assessed by determining the count of CD31‐ and vWF‐labelled endothelial cells (EC) in five areas from each liver section at 200 × magnification and is expressed as the number of CD31‐ or vWF‐positive vessels per field 7, 14. Every CD31‐ or vWF‐positive EC or EC cluster that was clearly separated from adjacent microvessels was counted as a single countable vessel.

### Western blot and quantitative real‐time RT‐PCR

Western blot and quantitative real‐time RT‐PCR were performed as described previously 7, 25, 30 and provided in the Appendix S1.

### Statistical analysis

Data are presented as mean ± S.D. Comparisons between two independent groups were performed using a two‐sample *t*‐test. Comparisons between multiple groups were performed by one‐way analysis of variance (anova) with *post hoc* Tukey's multiple comparison tests or by two‐tailed unpaired Student's *t*‐tests. Statistical analyses were performed using GraphPad Prism 7 software (La Jolla, CA, USA). Statistical significance is indicated as follows: *P < 0.05, **P < 0.01 and ***P < 0.001; ‘NS’ indicates not significant.

## Results

### PlGF expression is up‐regulated in the CCl_4_‐induced rodent model of liver cirrhosis as well as in patients with cirrhosis

**Figure 2:**
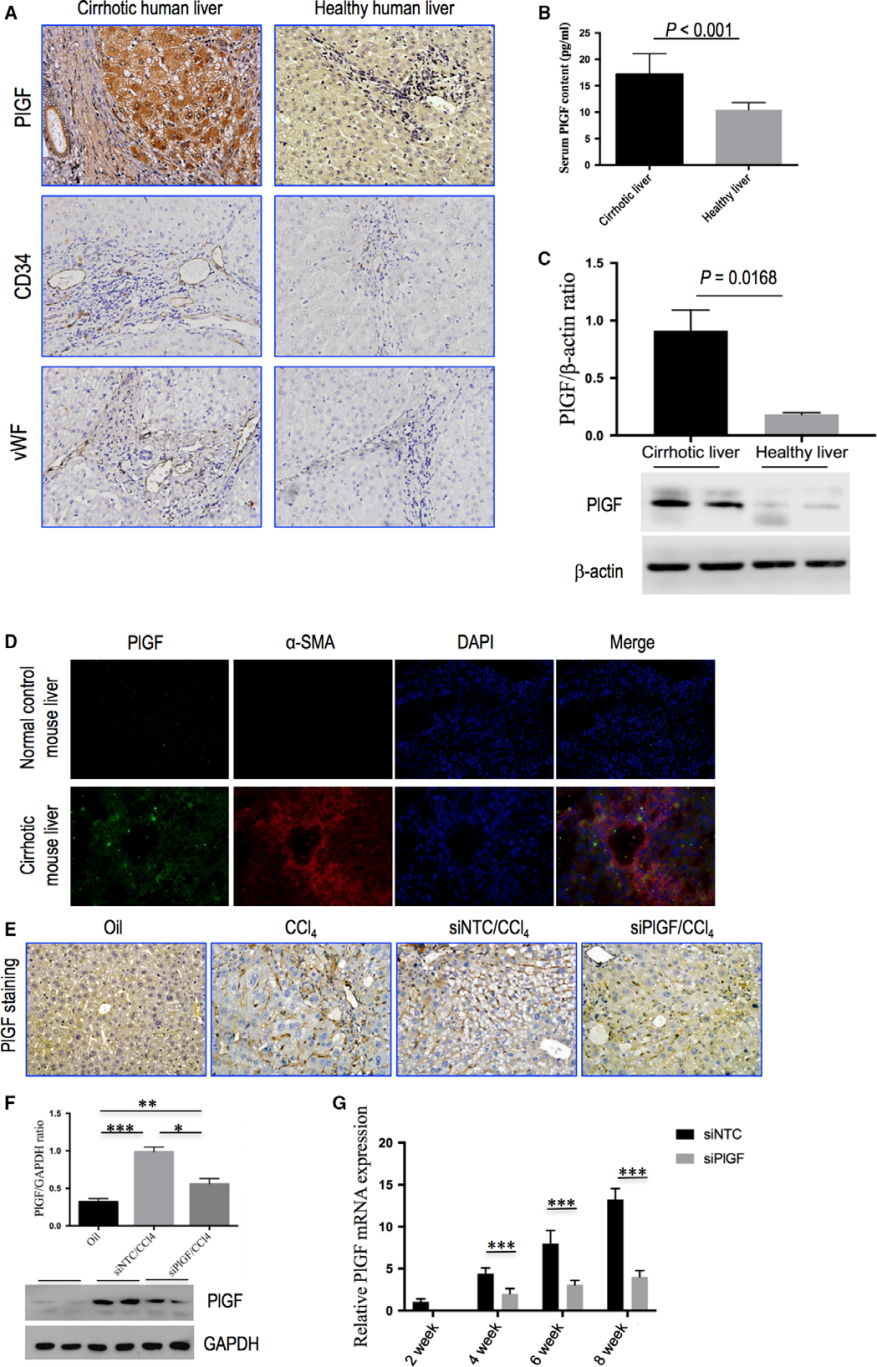
PlGF expression is up‐regulated in the CCl_4_‐induced rodent model of liver cirrhosis as well as in patients with cirrhosis, and PlGF in fibrotic liver is suppressed by system delivery *in vivo* PlGF siRNA. **(A)** Immunohistochemical staining of PlGF, CD34 and vWF in human liver samples (original magnification: ×200). **(B)** Serum PlGF contents examined by ELSA. **(C)** Western blot analysis of human cirrhotic PlGF expression and β‐actin as loading control (*n* = 3). **(D)** Representative images of double immunofluorescence of PlGF and α‐SMA in livers from each group of mice. Cell nuclei (blue) fluorescent staining was performed using DAPI. Colocalization of α‐SMA (red) and PlGF (green) is shown in merge panel. Original magnification: ×400. **(E)** Representative microscopy images of PlGF immunohistochemistry in livers from each group of mice (original magnification: 200×). **(F)** Western blotting analysis of PlGF expression in lysed liver tissue of each group at 8 weeks. Fibrotic mice treated with PlGF siRNA (siPlGF) or NTC siRNA (siNTC), with results normalized relative to the expression of GAPDH (*n* = 3). **(G)** Quantitative RT‐PCR comparing relative levels of PlGF mRNA expression in liver tissues following 2, 4, 6, and 8 weeks of CCl_4_ administration. The expression was normalized against GAPDH. Gene expression folds are presented as fold increase over 2‐week mice (before siRNA injection).

In established human liver fibrosis, regardless of aetiology (hepatitis B or C, autoimmune, alcohol‐induced or primary biliary cirrhosis), PlGF expression was undetectable in control normal liver and dramatically increased in the cirrhotic nodules of hepatocytes and non‐parenchymal cells, particularly at the portal tracts and fibrous septa, which was partly associated with neovessels in the hepatic scar as shown by immunohistochemistry (IHC) (Fig. 2A). We found that serum PlGF levels are elevated in patients with cirrhosis compared with those in healthy control individuals (17.16 ± 3.89 *versus* 10.48 ± 1.34 pg/ml, *P* < 0.001; Fig. 2B). Additionally, as shown in Figure 2(C), there was an approximately fivefold increase in PlGF protein levels in cirrhotic human livers compared with health control livers.

Moreover, PlGF expression was examined in a well‐established mouse model of liver fibrosis induced by CCl_4_, and we found PlGF expression and distribution in fibrotic mice as similarly as human samples (Fig. 2D and E). As shown in Figure 2(D), in fibrotic mice liver, PlGF immunofluorescence colocalized with α‐SMA and in cells located hepatic sinusoids, suggesting that PlGF expression is up‐regulated in pro‐fibrotic myofibroblasts. Furthermore, increased hepatic PlGF protein expression was also confirmed by Western blot analysis of cirrhotic mice induced following 8‐week CCl_4_ injection (Fig. 2F).

### PlGF is overexpressed in activated HSCs and its expression is induced by hypoxia dependent on HIF‐1α

**Figure 3:**
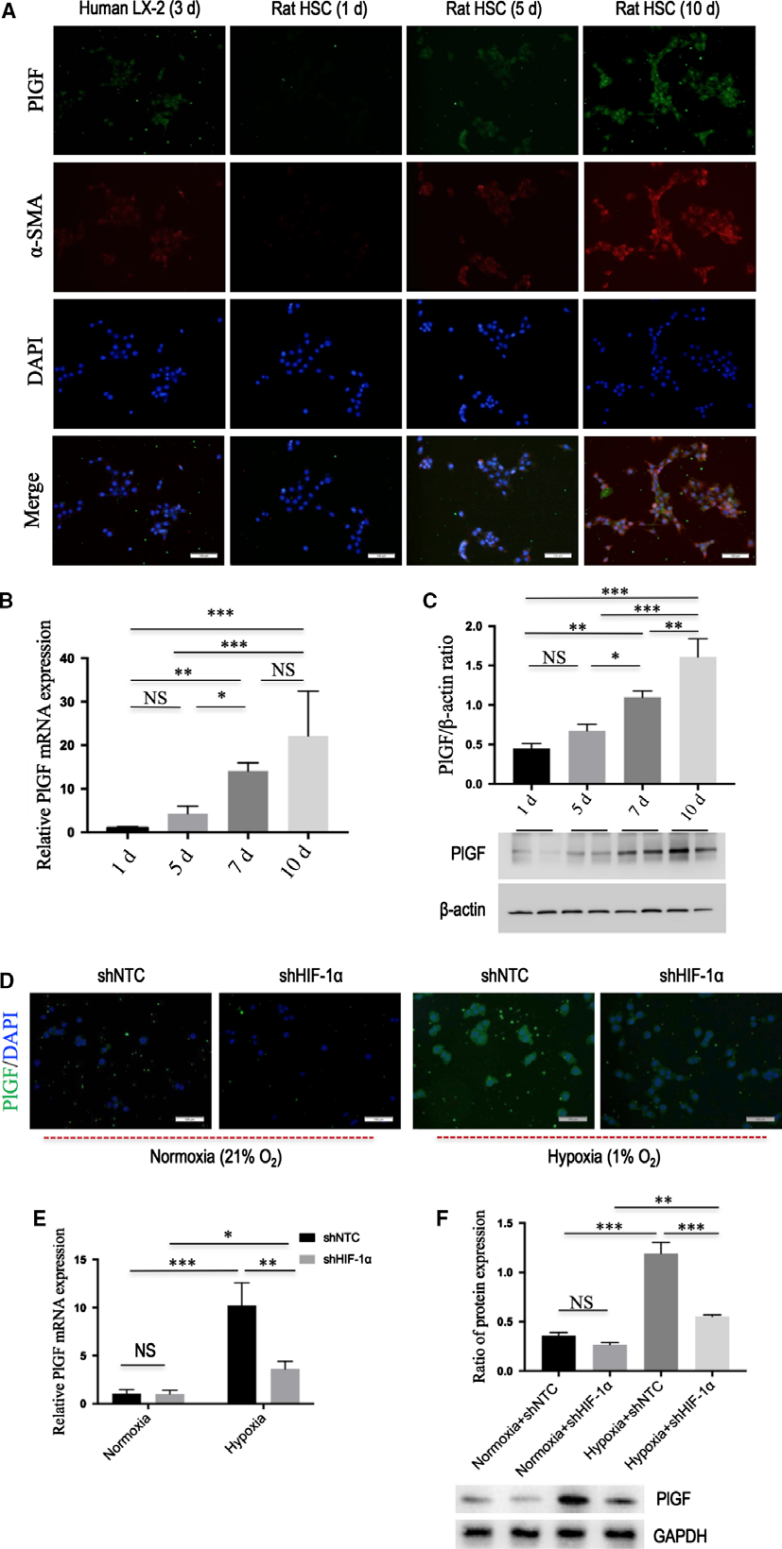
PlGF is overexpressed in activated hepatic stellate cells (HSCs) and its expression is induced by hypoxia dependent on HIF‐1α. **(A)** Immunofluorescent expression of PlGF (green) and α‐SMA (red) in LX‐2 human cell line and primary rat HSCs at different times *in vitro* activation; DAPI as blue nuclear counterstain. Scale bar = 100 μm for each picture. **(B)** The levels of PlGF mRNA expression in rat HSCs at different times *in vitro* activation were examined by quantitative RT‐PCR (*n* = 5). **(C)** The levels of PlGF protein expression in rat HSCs at different time points *in vitro* activation were examined by Western blot. **(D)** Immunofluorescent expression of PlGF (green) in rat HSCs; DAPI as blue nuclear counterstain. After infection with HIF‐1α shRNA (shHIF‐1α) or NTC shRNA (shNTC), cells were cultivated under hypoxic conditions (1% O_2_) or normoxia (21% O_2_) for 24 hrs. Scale bar = 100 μm for each picture. **(E)** The levels of PlGF mRNA expression in rat HSCs were measured by quantitative RT‐PCR. **(F)** Western blot analysis demonstrating effective silencing of HIF‐1α on the expression of PlGF in rat HSC, and GAPDH as loading control (*n* = 3).

In agreement with the results obtained in cirrhosis of rodent and human liver, we demonstrated that PlGF was highly expressed in both LX‐2 cells and primary rat HSCs by immunofluorescence staining (Fig. 3A). We also observed that PlGF staining increased during primary HSCs culture‐dependent development into myofibroblast‐like cells (Fig. 3A). Furthermore, we analysed the presence of PlGF gene expression changes during HSC activation in rat‐derived primary cultures of HSCs, and there was a significant increase in the levels of PlGF mRNA during their activation as shown in Figure 3(B). Similarly, the activation of HSCs is paralleled by an increase in PlGF protein expression (Fig. 3C). Additionally, we simultaneously examined the main PlGF receptors VEGFR‐1, VEGFR‐2 and neuropilin‐1 (NRP‐1) expression by quantitative RT‐PCR, the results showed that VEGFR‐1 and NRP‐1 mRNA increased as HSC activation, but the level of VEGFR‐2 mRNA expression is constant the same as control (Fig. S2).

Hypoxia is believed to be a pro‐fibrotic stimulus that contributes to the development of fibrosis in multiple ways; which can directly regulate gene transcription *via* binding of HIF‐1α 5, 6, 31.To assess whether hypoxia could induce PlGF expression in HSCs dependent on HIF‐1α. First, primary rat HSCs were subjected to hypoxia (1% O_2_) for 24 hrs, and we found that hypoxia obviously induced PlGF mRNA and protein expression (Fig. 3D–F), and associated with the increased expression of HIF‐1α in HSCs (Fig. S1). Next, primary rat HSC was transfected with HIF‐1α shRNA and then was challenged with hypoxia (1% O_2_) for 24 hrs, which showing that HIF‐1α knockdown effectively blocked PlGF expression (Fig. 3D–F). Together, the results suggested hypoxia‐induced PlGF expression in HSCs is dependent on HIF‐1α signalling.

### PlGF in fibrotic liver is suppressed by system delivery *in vivo* PlGF siRNA

In this study, we used a chemically synthesized short, double‐stranded RNA having well‐defined structure with a phosphorylated 5′ end and hydroxylated 3′ ends with two overhanging siRNA to target hepatic PlGF expression. After administration of CCl_4_ for 2 weeks, we gave PlGF siRNA four cycles by injection (Fig. 1). IHC staining revealed that intrahepatic PlGF‐positive cells were increased and remarkable at 8 weeks, and the staining located in the non‐parenchymal cells and hepatocytes around the accumulated fibrotic area. However, weak staining signal was observed in the livers treated with PlGF siRNA (Fig. 2E). Consistent with our histological findings, Western blotting results showed that liver PlGF expression was significantly reduced by the specific PlGF siRNA therapy (Fig. 2F). Additionally, to further ascertain the effect of siRNA‐mediated suppression of PlGF expression *in vivo*, we analysed liver PlGF mRNA levels at 0, 2, 4 and 6 weeks after siRNA delivery. Our results revealed that the levels of PlGF mRNA expression were gradually increased after CCl_4_ injection, which were significantly down‐regulated at their corresponding time‐points by PlGF siRNA administration (Fig. 2G).

### Knockdown of hepatic PlGF reduces liver injury, liver inflammation, and intrahepatic macrophage recruitment in mice induced by CCl_4_

**Figure 4:**
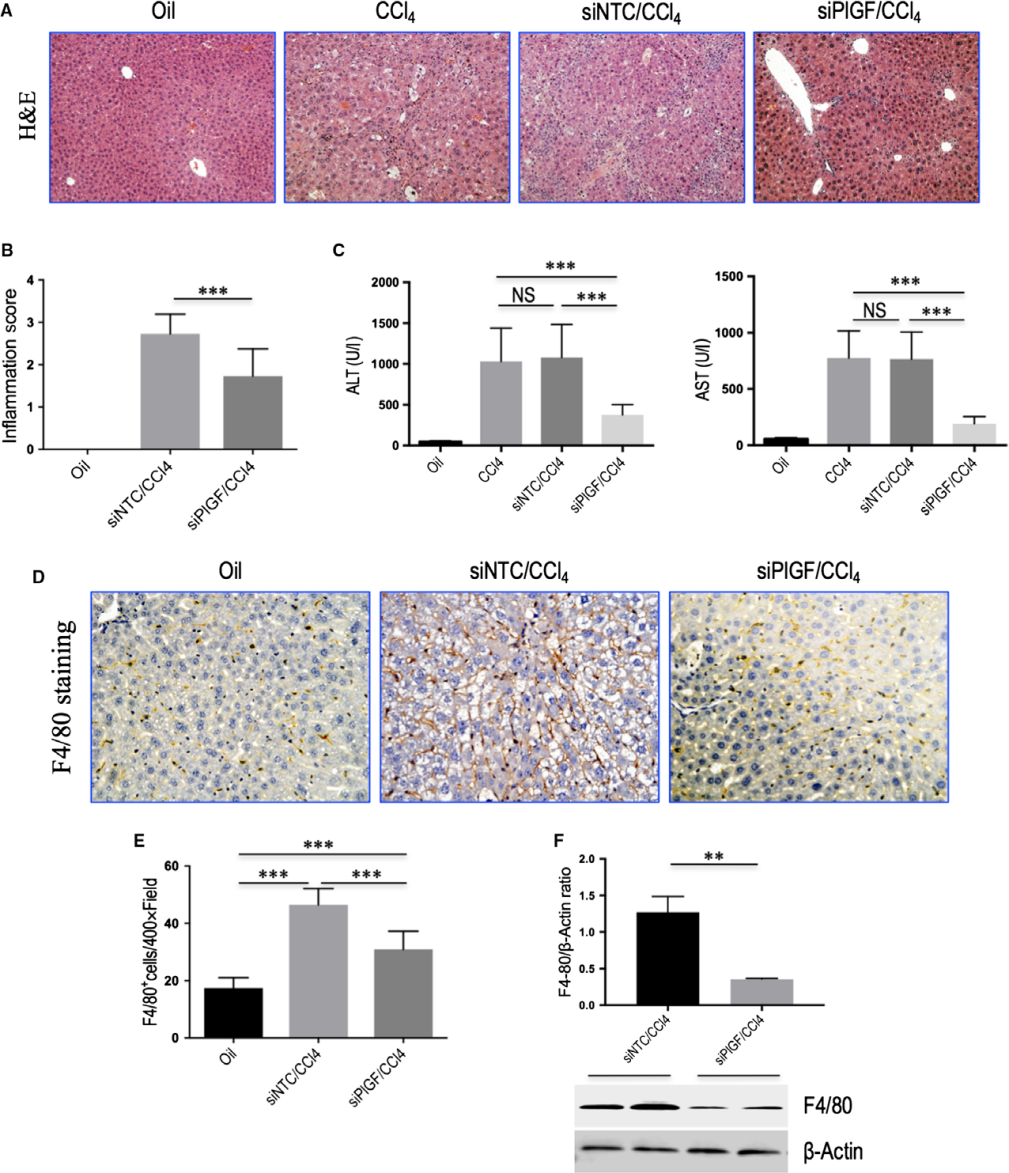
Knockdown of hepatic PlGF reduces liver injury, liver inflammation, and intrahepatic macrophage recruitment in mice induced by CCl_4_. **(A)** Histological images of livers stained with H&E. Original magnification: ×100. **(B)** Inflammation scores. **(C)** Serum ALT and AST concentrations in mice from each group (*n* = 6/group). (D) Representative microscopy images of F4/80 immunohistochemistry (original magnification: ×200). **(E)** Quantization of F4/80‐positive macrophages. Results mean of five fields each section and *n* = 6/group. **(F)** Western blotting analysis of F4/80 expression in lysed liver tissues, with results normalized relative to the expression of β‐actin (*n* = 3).

**Figure 5:**
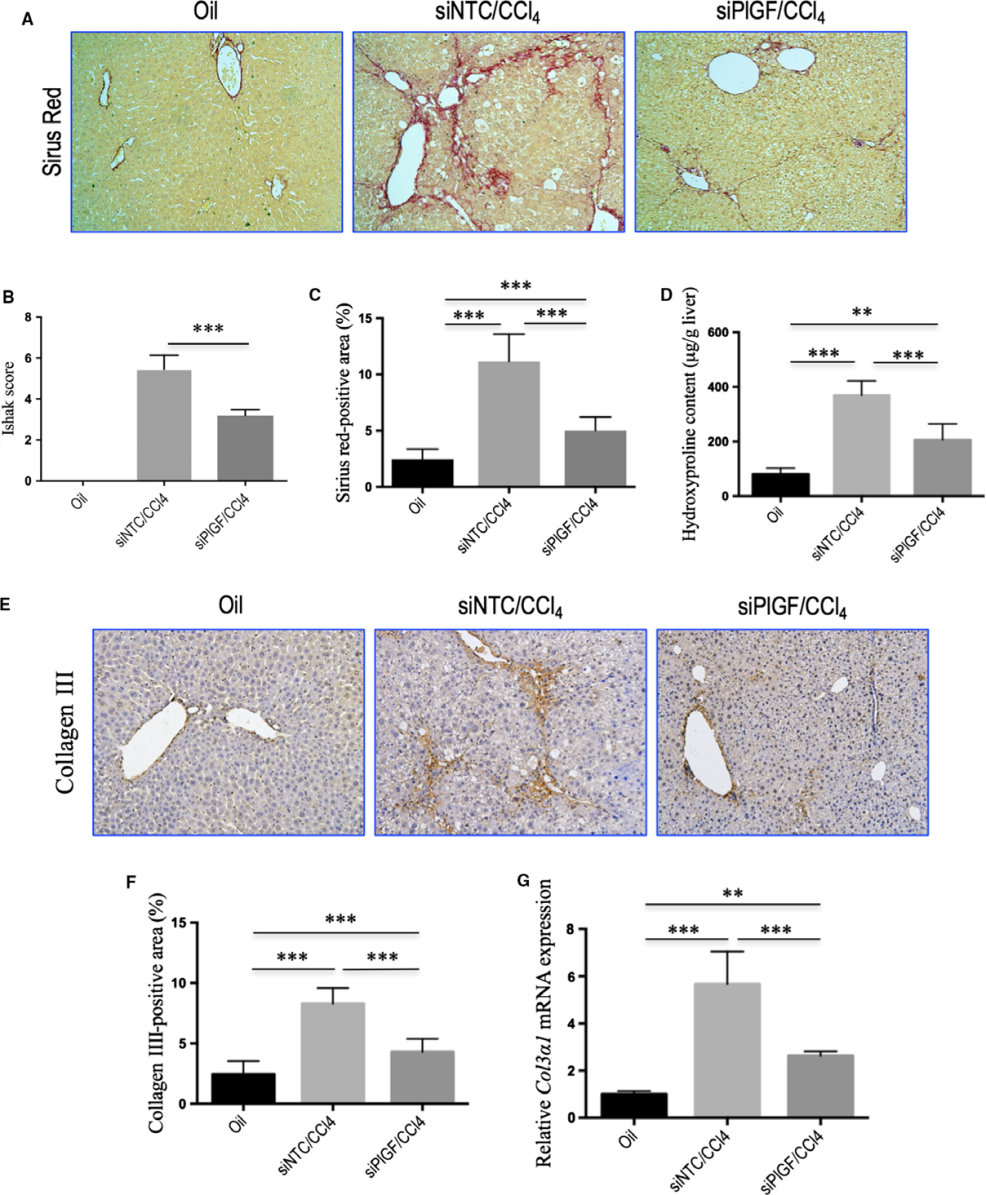
Knockdown of hepatic PlGF attenuates CCl_4_‐induced liver fibrosis in mice. **(A)** Sirius red staining of hepatic sections (original magnification: ×100). **(B)** Assessment of liver fibrosis based on Scheuer's scoring system. **(C)** Hepatic fibrotic area based on Sirius red staining (*n* = 10/group). **(D)** Hepatic hydroxyproline content. **(E)** Representative microscopy images of collagen III immunohistochemistry (original magnification: ×100). **(F)** Quantitative analysis of collagen III‐positive area by ImageJ software (NIH). *n* = 8/group. **(G)** Quantitative RT‐PCR results of *Col3*α*1* mRNA expression in liver tissue. Concentrations were normalized relative to GAPDH expression, and values are expressed as mean ± S.D. fold increase over oil‐treated control mice.

Histological studies have demonstrated that liver from CCl_4_‐treated mice receiving NTC siRNA exhibited marked architectural changes, with extensive deposition of fibrillar collagen and small regenerating nodules, notably, however, only mild architectural alterations in livers were noted in mice receiving PlGF siRNA knockdown (Figs 4A and 5A). In addition, the degree of hepatic inflammation in PlGF knockdown group decreased when compared with those of NTC siRNA‐treated group as shown in Figure 4(B) (2.73 ± 0.47 *versus* 1.73 ± 0.65, respectively, *P* = 0.0005). This is indeed also supported by the findings that serum AST and ALT levels were decreased by PlGF silencing to the mice following 8 weeks of CCl_4_ injection (Fig. 4C).

Based on the pivotal role of monocytes and macrophages in the progression of liver inflammation and fibrosis, we investigated whether suppression of PlGF expression affected inflammatory cells infiltration. Indeed, our IHC results showed that a massive accumulation of F4/80‐positive macrophages could be observed in fibrotic livers in mice after injection with CCl_4_ for 8 weeks. Notably, however, the increase in macrophage recruitment associated with fibrosis was significantly reduced in the PlGF siRNA‐treated mice when compared with the NTC siRNA‐treated mice (Fig. 4D). The results were further confirmed by quantification of the F4/80 staining cells, suggesting that repeated CCl_4_ injection obviously increased the number of F4/80 staining cells per high‐power field (HPF) and that the increased number of F4/80 cells were significantly lower in PlGF siRNA‐treated fibrotic mice than in NTC siRNA‐treated mice (30.9 ± 6.3 *versus* 46.4 ± 5.7, *P* < 0.001; Fig. 4E). These results correlated with the decreased F4/80 protein expression in PlGF siRNA‐treated fibrotic mice (Fig. 4F). Taken together, these results suggested that siRNA‐mediated PlGF knockdown significantly reduced Kupffer cell recruitment to the liver of CCl_4_‐induced fibrotic mice.

To further understand the link between PlGF knockdown and the reduction in inflammatory infiltrate, the expression of pro‐inflammatory adhesive molecules such as C‐X‐C motif chemokine ligand 10 (CXCL10), intercellular adhesion molecule 1 (ICAM‐1), and C‐C motif chemokine ligand 2 (CCL2) in the vasculature of fibrotic mice was analysed. We found that the levels of CXCL10, CCL2, and ICAM‐1 mRNA expression in livers were markedly enhanced following 8 weeks CCl_4_ injection, but these increase in livers were attenuated by PlGF siRNA treatment (Fig. S3A). Meanwhile, in normal murine liver, weak constitutive expression of CXCL10, CCL2 and ICAM‐1 was observed on vascular and sinusoidal endothelial cells, and hepatocytes showed no expression (Fig. S3B). Expression of these chemokines was increased on vascular and sinusoidal endothelial cells in fibrotic liver; however, knockdown PlGF with siRNA decreased the expression signalling of those chemokine proteins in livers as shown in IHC study (Fig. S3B). These findings were supported by our Western blotting, demonstrating the increase in these chemokines in fibrotic livers was indeed attenuated by PlGF silencing (Fig. S3C).

### Knockdown of hepatic PlGF attenuates CCl_4_‐induced liver fibrosis in mice

As shown in Figures 4(A) and 5(A), following 8 weeks of CCl_4_ administration, mice developed remarkable fibrosis showing the characteristic pattern of perivenular and periportal deposition of connecting tissue with development of thin septa, architectural distortion, and bridging fibrosis. Few areas of healthy hepatocytes were present. However, RNAi‐mediated PlGF knockdown mice exhibited thinner septa, mild portal or pericellular fibrosis of the liver, and more preserved hepatic parenchyma after 8 weeks of CCl_4_ injection. As revealed by the histological analysis of liver sections, there was a lower mean fibrosis score in mice receiving PlGF siRNA treatment compared with those of the mice receiving NTC siRNA treatment (3.18 ± 0.98 *versus* 5.46 ± 0.69, *P* < 0.001; Fig. 5B). This was confirmed by Sirius red‐stained area analysis showing a significant reduction in the percentage of fibrosis area in PlGF siRNA‐treated fibrotic mice compared to those of the NTC siRNA‐treated fibrotic mice (Fig. 5C).

Hepatic hydroxyproline content was also significantly lower in PlGF siRNA‐treated mice than in NTC siRNA‐treated mice (Fig. 5D). Additionally, IHC evaluation showed that the deposition of collagen III was also increased in the portal tracts, septa and perisinusoidal spaces, primarily in the periportal zones of the lobules in mice treated with NTC siRNA, whereas PlGF siRNA treatment attenuated collagen III accumulation in livers (Fig. 5E). These findings were supported by quantification of collagen III‐positive areas showing a decrease the areas by 48.2% in PlGF siRNA administered to fibrotic mice compared with NTC siRNA‐treated animals (Fig. 5F). In addition, we also examined the expression of *Col3a1* mRNA, which encodes collagen III in liver tissue, which suggesting the levels of intrahepatic *Col3*α*1* mRNA expression were down‐regulated by siRNA‐mediated PlGF knockout in fibrotic mice (Fig. 5G). Overall, these results suggested that PlGF silencing led to a significant reduction of liver fibrosis in mice induced by CCl_4_.

### Knockdown of hepatic PlGF attenuates hepatic angiogenesis and inhibits pro‐angiogenic factors in mice with liver fibrosis

**Figure 6:**
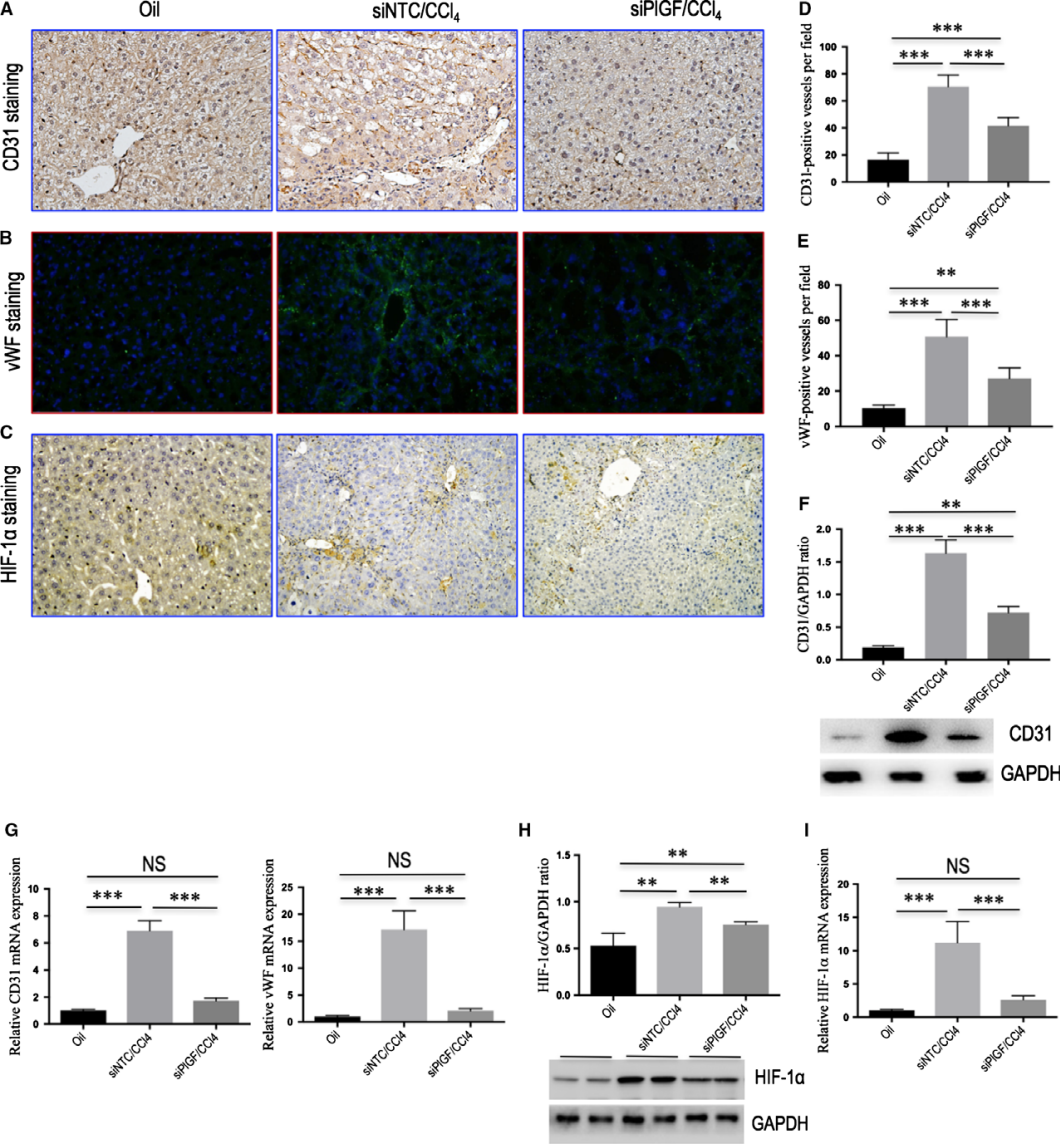
Knockdown of hepatic PlGF attenuates hepatic angiogenesis and inhibits pro‐angiogenic factors in mice with liver fibrosis. **(A‐C)** Representative images of CD31 **(A)** and HIF‐1α **(C)** immunohistochemistry staining, and vWF immunofluorescence staining **(B)** in the liver of mice from each group (original magnification: ×200). **(D, E)** Microvessel density (MVD) was assessed by counting CD31‐positive vessels or vWF‐positive vessels (*n* = 10/group). **(F)** Western blot analysis of hepatic CD31 expression and GAPDH as loading control. **(G)** Hepatic CD31 and vWF mRNA expression were determined by quantitative RT‐PCR, and results were normalized to GAPDH mRNA (*n* = 6/group). **(H)** Western blotting analysis of HIF‐1α expression in lysed liver tissues, with results normalized relative to the expression of GAPDH. **(I)** Hepatic expression of HIF‐1α mRNA by quantitative RT‐PCR, and results were normalized to GAPDH mRNA (*n* = 6/group).

We also conducted studies to determine hepatic neovascularization and the expression of angiogenic factors in livers from each group of mice. The endothelial cell marker CD31 was expressed in the endothelium of the veins and in the central veins in normal livers, but not along the sinusoids (Fig. 6A). The administration of CCl_4_ for 8 weeks led to a significantly increased number of CD31‐positive vessels in NTC siRNA‐treated mice (Fig. 6A), with a mean microvessels density (MVD) 70.6 ± 8.6/HPF (Fig. 6D). Knockdown of PlGF significantly attenuated CCl_4_‐induced hepatic angiogenesis in mice, as demonstrated by the reduction in MVD to 41.6 ± 6.0/HPF (Fig. 6D). These results were further supported by the levels of CD31 mRNA and protein expression in livers; showing that were inhibited by PlGF silencing (Fig. 6F and G). A similar histological pattern was observed in the expression of vWF, another endothelial cell marker, indicating up‐regulated in livers from CCl_4_‐injected mice with NTC siRNA treatment (Fig. 6B and E); however, PlGF siRNA treatment decreased the expression of vWF signalling and the number of vWF‐positive vessels within the liver (Fig. 6E) as well as vWF gene expression (Fig. 6G). Together, these results indicated that PlGF silencing prevented the fibrosis‐associated angiogenesis.

Moreover, IHC staining demonstrated that HIF‐1α obviously up‐regulated in fibrotic livers compared with those of oil‐treated controls, while PlGF siRNA‐treated mice had a weak expression signalling in livers compared with those of NTC siRNA‐treated mice (Fig. 6C). Western blot demonstrated significantly reduced protein expression of HIF‐1α in PlGF siRNA‐treated fibrotic mice compared to those of the NTC siRNA‐treated mice (*P <* 0.01; Fig. 6H). Similarly, the gene levels of HIF‐1α expression in fibrotic liver were also inhibited by treatment with PlGF siRNA *in vivo* (Fig. 6I). PlGF exclusively binds to VEGFR‐1 and not VEGFR‐2 32, and we therefore examined VEGFR‐1 expression; and our Western blotting confirmed that VEGFR‐1 expression was increased in the fibrotic livers, whereas PlGF silencing decreased the levels of VEGFR‐1 expression (Fig. S4). In addition, we also assayed the intrahepatic expression of VEGF, VEGFR‐1, VEGFR‐2 and NRP‐1 mRNAs by quantitative RT‐PCR. Compared to the NTC siRNA‐treated fibrotic mice induced by CCl_4_, PlGF siRNA treatment significantly down‐regulated intrahepatic VEGF, VEGFR‐1 and NRP‐1 mRNA gene expression, while VEGFR‐2 mRNA levels were unchanged (Fig. S4).

### Knockdown of hepatic PlGF inhibits HSC activation in CCl_4_‐induced liver fibrosis in mice

**Figure 7:**
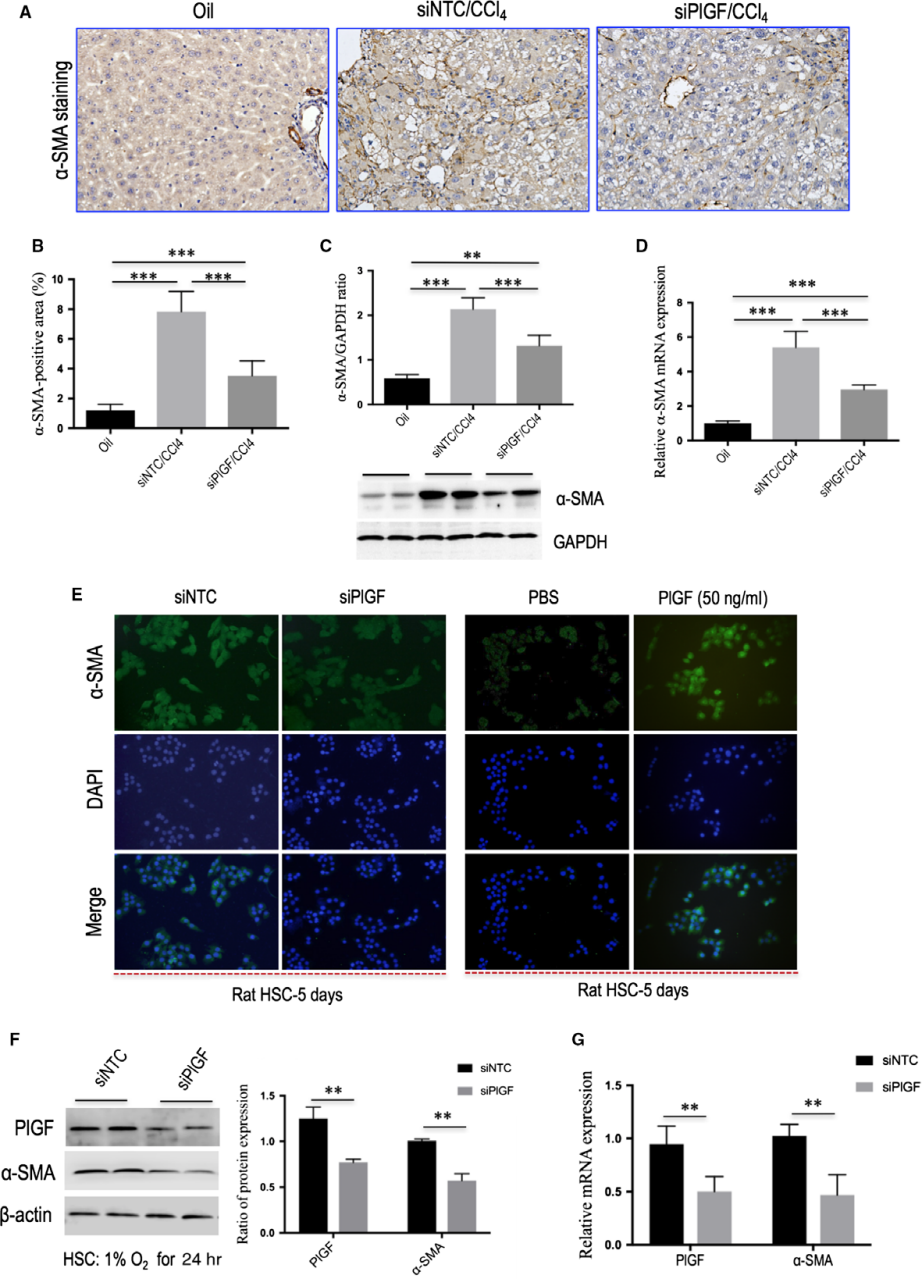
Knockdown of hepatic PlGF inhibits hepatic stellate cells (HSC) activation in CCl_4_‐induced liver fibrosis in mice and *in vitro*. **(A)** Representative images of α‐SMA immunohistochemistry in the liver of mice from each group (original magnification: ×200). **(B)** Quantification of α‐SMA‐positive area (%) by ImageJ software (NIH). **(C)** Western blot analysis of hepatic α‐SMA expression and GAPDH as loading control. **(D)** Hepatic α‐SMA mRNA expression was determined by quantitative RT‐PCR, with results were normalized relative to the expression of GAPDH (*n* = 6). **(E)** Immunofluorescence for α‐SMA (green) in primary rat HSCs; DAPI as blue nuclear counterstain. Cells were challenged with hypoxia (1% O_2_) for 24 hrs after transfection with PlGF siRNA or NTC siRNA, or were stimulated with rPlGF (50 ng/ml) for 48 hrs, respectively. **(F)** Knockdown of PlGF using siRNA in rat HSCs reduced α‐SMA expression examined by Western blot. **(G)** The mRNA levels of α‐SMA and PlGF were determined by quantitative RT‐PCR, with results were normalized relative to the expression of GAPDH (*n* = 5).

Except that activated HSCs are considered central ECM‐producing cells within the injured liver 2, HSCs are also being increasingly recognized for their role in angiogenesis and vascular remodelling 5, 6, 12. To determine whether PlGF knockdown inhibited the activity of HSCs, liver sections were immunostained with antibody to α‐SMA, and we observed relatively weak intensity in PlGF siRNA‐treated fibrotic mice compared to those in NTC siRNA‐treated mice (Fig. 7A). Moreover, computer‐assisted semiquantitative analysis demonstrated that the number of α‐SMA‐positive cells was significantly lower in fibrotic livers from PlGF siRNA‐treated mice than in those from NTC siRNA‐treated mice (3.52 ± 1.01% *versus* 7.83 ± 1.36%, *P* < 0.001; Fig. 7B). These findings were substantiated by quantitative RT‐PCR and by Western blotting experiments, which suggesting the levels of α‐SMA mRNA and protein expression were reduced following PlGF knockdown (Fig. 7C and D). These *in vivo* findings indicated that PlGF knockdown efficiently inhibited the activation of HSCs in damaged livers.

### PlGF knockdown by siRNA inhibits the proliferation and activation of HSCs *via* the PI3K/Akt signalling pathway

**Figure 8:**
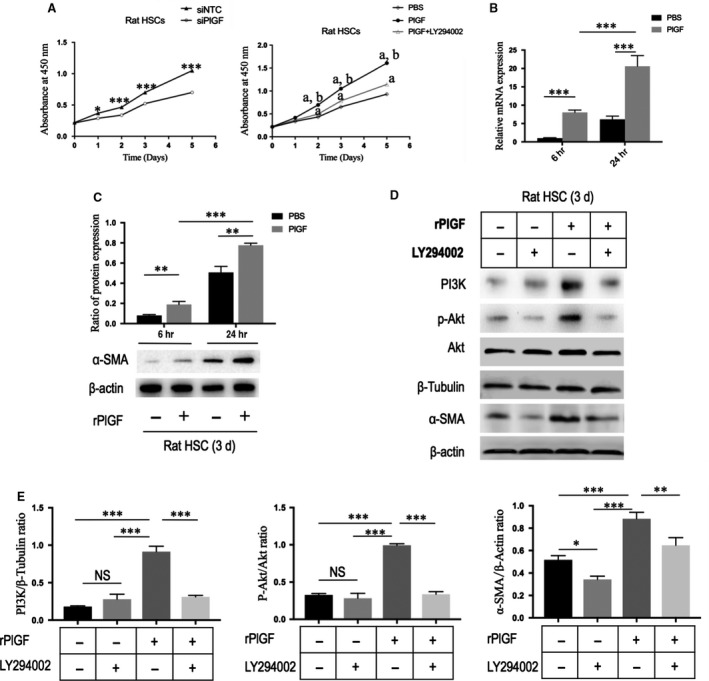
PlGF knockdown by siRNA inhibits the proliferation and activation of hepatic stellate cells (HSCs) *via* the PI3K/Akt signalling pathway. **(A)** Measurement of cell proliferation of rat HSCs using CCK‐8 assay. Cells were transfected with PlGF siRNA or NTC siRNA; cells were treated with rPlGF administration (50 ng/ml) or co‐incubation with PI3K inhibitor LY294002 for 5 days. ^a^*P* < 0.001 compared with mimics control (PBS), ^b^*P* < 0.001 compared with PlGF+LY294002. **(B)** The mRNA levels of α‐SMA in rat HSCs with stimulation PlGF (50 ng/ml) for 6 or 24 hrs. **(C)** Representative Western blot of α‐SMA expression in rat HSC treated with rPlGF (50 ng/ml) or PBS for 6–24 hrs and quantification compared to β‐actin content. **(D)** Western blot for PI3K, phospho‐Akt (p‐Akt), Akt and α‐SMA in rat HSCs, and β‐tubulin or β‐actin as loading controls. Cells were treated with rPlGF administration (50 ng/mL) or co‐incubation with PI3K inhibitor LY294002 for 8 hrs. **(E)** The Western blot results of part **(D)** were quantified by densitometry.

In order to tested whether PlGF is required for HSC activation and proliferation *in vitro*. Firstly, we examined the effect of knockdown of PlGF in HSCs with siRNAs on the cells function. After rat HSCs were isolated and cultured for 3 days, cells were transfected with PlGF siRNA or NTC siRNA, and then challenged with hypoxia for 24 hrs. We found genetic silencing of PlGF in primary rat HSCs decreased the levels of α‐SMA mRNA and protein *in vitro* (Fig. 7E–G). In addition, the proliferation of HSCs was significantly inhibited by PlGF‐specific siRNA as determined by CCK‐8 assays (Fig. 8A).

Next, we tested if recombinant rat PlGF (rPlGF) may increase HSC proliferation and activation, and we observed enhanced levels of α‐SMA in HSC upon stimulation with rPlGF (50 ng/ml) for up to 48 hrs as detected by mRNA (Fig. 8B) and protein expression (Figs 7E and 8C). Furthermore, we found that rPlGF treatment induced the increase expression of α‐SMA protein in HSCs in a dose‐dependent manner (Fig. S5). Additionally, we also observed that rPlGF (50 ng/ml) induced proliferation rates both in human LX‐2 cell (Fig. S6) and in rat primary HSC (Fig. 8A).

Finally, to gain insight into the mechanism underlying the effect of PlGF on HSCs functions, we examined the changes in signalling transduction pathway plausibly involved in mediating PlGF action. As phosphatidylinositol 3‐kinase (PI3K)/Akt signalling pathway has been shown to regulate aspects of HSC activation *in vitro*, including proliferation, survival and collagen synthesis 33, 34, 35, we focused on exploring the effects of rPlGF on this signalling pathway. As shown in Figure 8(D) and (E), the result showed that rPlGF treatment effectively induced the phosphorylation of Akt (p‐Akt) and PI3K expression in rat HSCs. Moreover, HSCs were treated with rPlGF in the absence or presence of the PI3K inhibitor, LY294002. Obviously, the Western blotting results showed that the p‐Akt and PI3K in rat HSC were significantly inhibited by co‐incubation with LY294002, thus influencing PlGF‐mediated effects on HSCs as shown by the cell proliferation rates and the change in α‐SMA expression (Fig. 8). Collectively, these results showed that the participation of PlGF on the HSC activation and proliferation *via* activation the PI3K/Akt signalling pathways.

## Discussion

In this work, we show the importance of PlGF in the development of liver fibrosis and hepatic angiogenesis in rodent models and in activation and proliferation of HSCs *in vitro*. Furthermore, we report that siRNA‐mediated down‐regulation of PlGF ameliorates liver fibrosis, inflammation and angiogenesis, and inhibits activation of HSCs both *in vivo* and *in vitro*. Importantly, these findings may provide a new insight for understanding the mechanism of PlGF contributing to liver fibrosis and angiogenesis.

Recently, much attention has been focused on PlGF, which is more important factor exclusively in pathologic angiogenesis 17, 18, 19, 20, 21, 22, 23, 24. Therefore, it has been suggested that PlGF blockade could inhibit these diseases processes without affecting normal health 17, 18, 19. In this study, along with elevated serum PlGF levels in patients with cirrhosis (Fig. 2B), we also found that hepatic PlGF expression was markedly increased in rodent models and patients with cirrhosis (Fig. 2); these results agree with the findings of others 21, 22. Moreover, our immunostaining studies further demonstrated that activated HSCs were the main cells expressed PlGF in fibrotic livers (Fig. 2D). Consistently with *in vivo* findings, *in vitro* experiments further demonstrated PlGF was overexpressed in both LX‐2 human cells and activated primary rat HSCs (Fig. 3). Notably, hypoxia has been shown to be a pro‐fibrotic stimulus that contributes to the development of fibrosis through an HIF‐mediated transcriptional response 5, 6, 25. Here, we observed that hypoxia could induce PlGF expression accompanying HIF‐1α activation in HSCs; however, HIF‐1α knockdown in HSCs abrogated hypoxia‐induced PlGF expression, indicating that these effects were HIF‐1α‐dependent (Fig. 3D–F). Similarly, we also demonstrated that HIF‐1α was increased in fibrotic livers *in vivo* (Fig. 6), which was consistent with previous reports 5, 6; however, PlGF‐specific siRNA inhibited the expression of HIF‐1α in fibrotic livers (Fig. 6), thus leading to the decreased liver fibrosis and angiogenesis. Of note, besides the regulation of genes involved in fibrogenesis, hypoxia and HIF‐1α have been shown to regulate target genes involved in vascular biology 10, 25.

PlGF‐induced angiogenesis may contribute to wound‐healing responses including liver fibrosis. In accordance with the present results, previous studies have demonstrated that hepatic VEGF, PlGF, VEGFR and NRP‐1 were up‐regulated in liver fibrosis 7, 22, 23. However, PlGF silencing significantly decreased the intrahepatic VEGF, VEGFR‐1 and NRP‐1 expression in CCl_4_‐treated fibrotic mice (Fig. S4). These data suggest that PlGF signalling pathway may trigger the microvascular proliferation associated with liver fibrogenesis, thereby contributing to the remodelling of liver architecture.

Inflammation is an important and complex feature of liver fibrosis as suggested by its role in the activation of HSCs. Moreover, inflammatory cell infiltration has often been linked to angiogenesis 10, 13, 36. Following liver injury, inflammatory cells are recruited in the injurious site through chemokine attraction 2, 36, 37. Of note, Kupffer cells not only activate HSCs but also stimulate the influx of bone marrow‐derived immune cells *via* release of CCL2 (also MCP‐1) and CCL5, driving fibrosis progression during chronic injury 2, 37. On the other hand, the recruitment process is also mediated by other chemokines and its receptors, such as CXCL10 and ICAM‐1 2, 36. Interesting, previous studies have shown that VEGFR‐1 is expressed on macrophages, and binding of PlGF to VEGFR‐1 stimulates the recruitment and/or activation macrophages leading to cancer‐associated angiogenesis 17, 19. Because PlGF itself acted as a strong chemoattractant for macrophages, and the siRNA‐mediated knockdown of PlGF has been shown to inhibit macrophages recruited in liver (Fig. 4D–F), suggesting a mechanism to explain the attenuated fibrosis‐associated angiogenesis. Additionally, the decreased expression of the pro‐inflammatory mediator CXCL10, CCL2 and ICAM‐1 in livers by PlGF silencing was also involved in the antifibrotic effect 36, 37. It should be noted that the CXC family of chemokines also operate in pathological angiogenesis preceding/perpetuating fibrosis 36.

In response to injury, quiescent HSCs proliferate and transdifferentiate into activated myofibroblasts, which are the main collagen‐producing cells during liver fibrogenesis 1, 2, 3, 4. In the present study, we found that PlGF played an important role in the activation and proliferation of HSCs, suggesting that PlGF could mediate effects on HSCs leading to liver fibrosis‐associated angiogenesis. On contrast, knockdown using PlGF siRNA suppressed the activation and proliferation of HSCs (Figs 7 and 8), which contributing to attenuate liver fibrosis and angiogenesis. We further demonstrated that the PlGF, which was induced by hypoxia during liver fibrosis dependent on HIF‐1α, was involved in HSC activation and proliferation through modulation of the PI3K/Akt signalling pathway (Fig. 8), while the addition of PI3K inhibitor to PlGF‐treated HSCs abrogated the proliferative and activated effects of PlGF. It is well known that PI3K/Akt signalling is necessary for HSC activation and proliferation 33, 34, 35. Thereby, our results indicated that PlGF induced activation and proliferation through PI3K/Akt signalling in HSCs. Our recent study demonstrated that PlGF induced PI3K/Akt phosphorylation in human intestinal microvascular endothelial cells (HIMECs) and pre‐treatment of PlGF‐stimulated HIMECs with LY294002 significantly inhibited the PlGF‐induced cell migration and tube formation 38. Based on our findings, it is reasonable to speculate that PlGF‐PI3K/Akt signalling pathways in HSCs are considered to be the potential therapeutic targets for antifibrosis.

However, it is important to mention that our study has some limitations. First, we examined the effect of PlGF on HSC activation and proliferation; perhaps it also directly influenced angiogenic activity 13, 20, 32. Second, given that PlGF is a secreted factor, its mainly released from activation of HSCs into surrounding, which may act on VEGFR‐1 or NRP‐1 expressed on liver sinusoidal endothelial cells (LSEC) and promote an increase in the pro‐angiogenic activity by a paracrine fashion 13, 24. Third, although this injection route delivers siRNA preferentially targeted to liver 39, 40, this is a challenging process and it is necessary to administer PlGF siRNA repeatedly for the continuous knockdown of PlGF mRNA *in vivo* in order to prevent the progression of hepatic fibrosis. Finally, the exact role of PlGF‐mediated angiogenesis in the fibrosis resolution is still not known and the potential benefit of its inhibition is incompletely characterized. Therefore, further studies on the current topic will need to be undertaken.

In conclusion, our study provides evidence that PlGF exerts strong fibrogenic and angiogenic effects in liver fibrosis, and siRNA‐mediated down‐regulation of PlGF ameliorates liver injury, inflammation, fibrosis and hepatic angiogenesis. The results highlights that inhibiting PlGF pathway might offer a novel therapeutic approach for chronic liver diseases associated with increased neoangiogenesis.

## Conflict of interest

The authors declare that they have no conflict of interests with the contents of this article.

## Supporting information


**Appendix S1** Experimental procedures.
**Figure S1** The transfected effective was confirmed by examined the gene and protein expression.
**Figure S2** The gene expression of VEGFR‐1, NRP‐1 and VEGFR‐2 during HSC activation in rat‐derived primary cultures of HSCs.
**Figure S3** PlGF knockdown attenuates the expression of proinflammatory adhesive molecules in the vasculature of fibrotic mice.
**Figure S4** Knockdown of hepatic PlGF inhibits pro‐angiogenic factors in mice with liver fibrosis.
**Figure S5** Recombinant PlGF (rPlGF) induced the increase expression of α‐SMA protein in primary rat HSCs in a dose‐dependent manner.
**Figure S6** The effect of PlGF on human LX‐2 cell line proliferation was determined using by CCK‐8 assay.
**Table S1** The synthesized oligos in the study *in vitro*.
**Table S2** Primer sequences used in this study.
