# Supplementary material for: Placental growth factor silencing ameliorates liver fibrosis and angiogenesis and inhibits activation of hepatic stellate cells in a murine model of chronic liver disease
Source: J Cell Mol Med. 2017 Apr 5;21(10):2370–85. doi: 10.1111/jcmm.13158 (PMC5618674; doi:10.1111/jcmm.13158)
Supplement: Supplementary file 1 — Appendix S1 Experimental procedures. Figure S1 The transfected effective was confirmed by examined the gene and protein expression. Figure S2 The gene expression of VEGFR‐1, NRP‐1 and VEGFR‐2 during HSC activation in rat‐derived primary cultures of HSCs. Figure S3 PlGF knockdown attenuates the expression of proinflammatory adhesive molecules in the vasculature of fibrotic mice. Figure S4 Knockdown of hepatic PlGF inhibits pro‐angiogenic factors in mice with liver fibrosis. Figure S5 Recombinant PlGF (rPlGF) induced the increase expression of α‐SMA protein in primary rat HSCs in a dose‐dependent manner. Figure S6 The effect of PlGF on human LX‐2 cell line proliferation was determined using by CCK‐8 assay. Table S1 The synthesized oligos in the study in vitro. Table S2 Primer sequences used in this study. [file JCMM-21-2370-s001.docx]

**Supplementary information accompanies this paper**

**PlGF silencing ameliorates liver fibrosis, angiogenesis and inhibits activation of hepatic stellate cells in a murine model of chronic liver disease**

Xi Li^1^, Hong-Chun Liu^2^, Qun-Yan Yao^2^, Qian-Wen Jin^2^, Bei-Li Xu^2^, Zheng Li^3^, Shun-Cai Zhang^2^, Chuan-Tao Tu^2*^

**Supplementary Experimental Procedures**

***Immunohistochemistry and Immunofluorescence***

The liver tissue sections were deparaffinized with xylene and rehydrated with graded ethanol. Antigen retrieval was performed by boiling the sections in citrate buffer for 10 min. The sections were stained according to routine immunohistochemistry procedures and visualized by means of a Vectastain ABC kit (Vector Laboratories). Slides were blocked with 5% goat serum and then incubated with primary antibodies overnight at **4℃** at the following concentrations: (1) anti-α-SMA antibody (1:100); (2) anti-Collagen-III antibody (1:150); (3) anti-PlGF, (1:200); (4) anti-vWF (1:100); (5) anti-HIF-1α antibody (1:150); (6) anti-CXCL10 (1:100); (7) anti-ICAM-1(1:150); (8) anti-VEGF (1:150); (9) anti-CD34 (1:100), and (10) anti-CD31(1:100). All antibodies were diluted in TBS containing 3% BSA. Negative-control antibodies consisted of species-matched and where appropriate, immunoglobulin G (IgG) subclass-matched Ig fractions, used at the same dilution as the secondary antibodies. The sections were subsequently washed with TBST and incubated with horseradish peroxidase (HRP)-conjugated goat anti-rabbit/mouse secondary antibodies, followed by incubation for 5 to 10 min with 3, 3′-diaminobenzidine tetrachloride and visualization of specific staining by light microscopy. Images were taken under a high-power field with a Leica DC500.

The dissected liver tissues retrieved were fixed in 4% paraformaldehyde solution for 30 min, washed with PBS (pH 7.4), embedded in optimum cutting temperature tissue compound (OCT compound, Sakura, Japan), and frozen at −80 °C for 1 day. Then the sections (10μm in thickness) were cut with a cryotome Cryostat (Leica, CM 1900, Germany) and placed on slides for immunofluorescence staining. Blocking was performed in PBS with 3 % BSA. The slides were incubated with antibody PlGF at the dilution of 1:100 overnight at 4℃, and subsequently incubated with antibody α-SMA at the dilution of 1:200 for 1 h at room temperature (RT). Alexa Fluor 594 donkey anti-mouse and Alexa Fluor 488 Donkey anti-Rabbit secondary antibodies (Yeasen Biotech, Shanghai, China) were incubated at 1:200 in PBS for 1 h at RT. After washing with Tris-buffered saline for three times, the Cell nuclei were counterstained with Dapi-Fluoromount-G^TM^ (SouthernBiotech, USA). Finally, the stained tissues were analyzed by fluorescence microscopy (BX51, Olympus, Japan).

For α-SMA and PlGF, rat HSCs or LX2 cells plated on 24-well plates and cultured on coverglass slips were fixed and permeabilized for 10 minutes in 4% paraformaldehyde, 0.2% TritonX-100 in PBS. Nonspecific binding was blocked with 3% BSA for 1h at RT, and then the cells were incubated with primary antibodies α-SMA (dilution 1:200) and PlGF (dilution 1:100) overnight at 4℃. After washing twice in PBS, the cells were incubated with fluorescein-labeled secondary antibody for 1h at RT in the dark. The nuclei were stained with DAPI in the dark for 40 min at RT. The slides were washed twice with PBS, covered with DABCO (Sigma-Aldrich, St. Louis, MO), and imaged by fluorescence microscopy (IX51, Olympus, Japan).

***Western blot***

Liver samples were homogenized (RIPA lysis buffer by adding protease inhibitor cocktail and phosphatase inhibitors) and centrifuged at 10,000 gat 4°C for 10 minutes. The protein concentration was measured using the Bicinchoninic Acid Protein Colorimetric Assay kits (BMI, Shanghai, China) with BSA as standard. Equivalent aliquots of protein samples (40 µg) were separated by electrophoresis on a 7.5-12% SDS-PAGE gel, and transferred onto polyvinylidenedifluoride membranes. The membrane was then incubated in blocking buffer (5% nonfat milk powder in TBST) for 3 h followed by incubation with primary antibody in TBST (100mM Tris-HCl, pH 7.5, 0.9% NaCl, 0.1% Tween 20) overnight at 4°C with the specific primary antibodies against PlGF, VEGFR-2, VEGFR-1, NRP-1, Collagen-III, F4/80, CD31, HIF-1α, α-SMA, PI3K, Akt, p-Akt, CXCL10, CCL2, and ICAM-1 (all 1:1000 dilution). The membrane was washed with TBST and then incubated with goat anti-rabbit, anti-mouse or anti-rat secondary antibodies (1:1500) for 2 h at room temperature. GAPDH, β-Tubulin and β-actin (1: 5,000 dilution) was used as internal control respectively. After washing off the unbound antibody with TBST, the expression of the antibody-linked protein was determined by an ECL^TM^ Western Blotting Detection Reagents (Amersham Pharmacia Biotech Inc., NJ, USA). The optical density of the bands was measured and quantified by ImageJ software (NIH, Bethesda, MD, USA). The optical density of protein products was expressed as arbitrary units in the figures.

***Total RNA isolation and quantitative real-time RT-PCR***

Total RNA was extracted from frozen liver tissues using Trizol reagent according to the protocol provided by the manufacturer (Life Technologies, Grand Island, NY). RNA extracts were reverse-transcribed with random hexamers and avian myeloblastosis virus reverse transcriptase using a commercial kit (Perfect Real Time, SYBR^®^ PrimeScriP^TM^TaKaRa, Japan). Real-time RT-PCR was performed for quantitative assessment of mRNA expression on an ABI Prism 7500 Sequence Detection system (Applied Biosystems, Tokyo, Japan) according to the manufacturer's protocol. Probes and primers for PlGF, VEGFR1, VEGFR2, NRP-1, VEGF, CD31, vWF, α-SMA, HIF-1α, type III collagen α1 (Col3α1), CXCL10, CCL2, ICAM-1 and GAPDH were purchased from Sangon Biotech Co., Ltd. (Shanghai, China, Supplementary Table1). The levels of target gene were normalized with respect to that of GAPDH for each sample using the ΔCt method. The relative differences in gene expression among study groups were determined using comparative Ct (ΔΔCt) method and fold expression was calculated using the formula 2^−ΔΔCt^, where ΔΔCt represents ΔCt values normalized with the mean ΔCt of control samples.

**Supplementary Table1.** Primer sequences used in this study

| Target gene | Species | Forward primers (5'-3') | Reverse primers (5'-3') |
| --- | --- | --- | --- |
| NRP-1 | Mouse | CCTGTGGTGGATTCTTCTC | CTGTCGGTATTGCTTGGTA |
| NRP-1 | Rat | TTGAACTTGTGGATGGTGTA | GCTTGTGTCTGTAGGTGAT |
| PlGF | Mouse | AACACAAGAAGCCTCCTAC | CATTCACAGAGCACATCCT |
| PlGF | Rat | AAGACAGCCAACATCACTAT | CATTCGCAGAGTACATCCT |
| VEGFR-1 | Mouse | GGCAGACCAATACAATCCTA | AGCGAGCAGACTTCAATG |
| VEGFR-1 | Rat | GACGGTTAGCACATTGGT | TCTCCTTCGGTTGGTATCT |
| HIF-1α | Mouse | CTGTCACGCTTCTGGGCCTGT | GCAGCAGGTGAGTGGGGCAT |
| HIF-1α | Rat | ACCTACCATCACTGTCACT | TTGTCTTCTGCTCCATTCC |
| vWF | Mouse | ATGGAGATGGCAGTGGAT | TGGCAGATGGTATGGAATG |
| α-SMA | Mouse | AGAACACGGCATCATCAC | GCAGTAGTCACGAAGGAAT |
| α-SMA | Rat | GCTGCTTCCTCTTCTTCC | GCTGCTTCCTCTTCTTCC |
| CD31 | Mouse | ACAGAGCCAGCAGTATGA | AATGACAACCACCGCAAT |
| VEGF | Mouse | GGCTGCTGTAACGATGAA | CTGCTGTGCTGTAGGAAG |
| Col3α1 | Mouse | CCTTCTACACCTGCTCCT | CTTCCTGACTCTCCATCCT |
| GAPDH | Mouse | TCTCCTGCGACTTCAACA | TGTAGCCGTATTCATTGTCA |
| GAPDH | Rat | GCTGCCTTCTCTTGTGACA | CCTTGACTGTGCCATTGAAC |
| CXCL10 | Mouse | GATGGATGGACAGCAGAG | GGAAGATGGTGGTTAAGTTC |
| CCL2 | Mouse | CAATGAGTAGGCTGGAGAG | GAAGTGCTTGAGGTGGTT |
| ICAM-1 | Mouse | CAGTGAGGAGGTGAATGTATA | GATGTGGAGGAGCAGAGA |
| F4/80 | Mouse | TTTTCAGATCCTTGGCCATC | ACACTGGGGCACTTTTGTTC |
| VEGFR-2 | Mouse | GCGAGACCATTGAAGTGA | GAAGGAGCCAGAAGAACAT |
| VEGFR-2 | Rat | GAACGCTTGCCTTATGATG | CTCTGACTGCTGGTGATG |
|  |  |  |  |

**Supplementary Table 2.** The synthesized oligos in the study in vitro.

| NO. | Accession | Target Sequence | CDS | GC% |
| --- | --- | --- | --- | --- |
| PlGF-siRNA  (26780-1) | NM_053595 | CCCTAATGAAGTGTCTCAT | 305..781 | 42.11% |
| PlGF-siRNA  (26781-1) | NM_053595 | TGGAAGTGGTGCCTTTCAA | 305..781 | 47.37% |
| PlGF-siRNA  (26782-1) | NM_053595 | GCCAACATCACTATGCAGA | 305..781 | 47.37% |
| NTC-siRNA | **-** | TTCTCCGAACGTGTCACGT | - | - |

**Supplementary Figures**

**Supplementary Figure S1**

**Figure S1.** The transfected effective was confirmed by examined the gene and protein expression. (A) The levels of HIF-1α mRNA expression by primary rat HSCs were measured by quantitative RT-PCR (n = 5). Concentrations were normalized relative to GAPDH expression. (B) Western blot analysis demonstrating effective target hepatic HIF-1α expression in primary HSC, and GAPDH as loading control (n = 3). **P* < 0.05; ***P* < 0.01; ****P* < 0.001.

**Supplementary Figure S2**

**Figure S2.** The gene expression of VEGFR-1, NRP-1 and VEGFR-2 during HSC activation in rat-derived primary cultures of HSCs. The levels of target gene expression in rat HSCs at different times *in* *vitro* activation were examined by quantitative RT-PCR (n = 5). Concentrations were normalized relative to GAPDH expression.

**Supplementary Figure S3**

**Figure S3.** PlGF knockdown attenuates the expression of proinflammatory adhesive molecules in the vasculature of fibrotic mice. (A)The levels of CXCL10, CCL2 and ICAM-1 mRNA expression in livers were determined by quantitative RT-PCR. Concentrations were normalized relative to GAPDH expression, and values are expressed as mean ± SEM fold increase over oil-treated control mice (*n* = 6/group). (B) Immunohistochemical staining for CXCL10, CCL2, and ICAM-1 in livers (original magnification 200×). (C) Western blotting analysis of CXCL10, CCL2 and ICAM-1 protein expression in lysed liver tissue from each group mice, with results normalized relative to the expression of GAPDH (*n* = 3). **P* < 0.05; ***P* < 0.01; ****P* < 0.001.

**Supplementary Figure S4**

**Figure S4.** Knockdown of hepatic PlGF inhibits pro-angiogenic factors in mice with liver fibrosis. (A) Western blotting analysis of VEGFR-1 expression in lysed liver tissue of siPlGF or siNTC treated CCl_4_ exposed mice, with results normalized relative to the expression of GAPDH (*n* = 3). (B) The intrahepatic expression of VEGF, VEGFR-1, VEGFR-2 and NRP-1 mRNAs by quantitative RT-PCR (n = 6). **P* < 0.05; ***P* < 0.01; ****P* < 0.001.

**Supplementary Figure S5**

**Figure S5.** Recombinant PlGF (rPlGF) induced the increase expression of α-SMA protein in primary rat HSCs in a dose-dependent manner. (A) Immunofluorescence for α-SMA (green) in primary rat HSCs; DAPI as blue nuclear counterstain. Cells were stimulated with different contents of rPlGF (25-100 ng/mL) for 24 h, respectively. (B) Western blotting for α-SMA in rat HSCs, with results normalized relative to the expression of GAPDH. Cells were treated with different contents of rPlGF (0-100 ng/ml).

**Supplementary Figure S6**

**Figure S6.** The effect of PlGF on human LX-2 cell line proliferation was determined using by CCK-8 assay. LX-2 Cells were cultivated with the indicated human recombinant PlGF (50 ng/mL) for 5 days.  ***P* < 0.01, ****P*< 0.001 compared with mimics control (PBS).
